# Use of Fourier Transform Infrared (FTIR) Spectroscopy to Study Cadmium-Induced Changes in *Padina Tetrastromatica* (Hauck)

**Published:** 2008-10-15

**Authors:** Lisette D’Souza, Prabha Devi, Divya Shridhar M.P., Chandrakant G. Naik

**Affiliations:** Bioorganic Chemistry laboratory, National Institute of Oceanography, Dona Paula Goa, 403 004, India

**Keywords:** fourier transform infrared (FTIR) spectroscopy, marine alga, Padina tetrastromatica, cadmium, stress-induced changes

## Abstract

The aim of this study is to adopt the approach of metabolic fingerprinting through the use of Fourier Transform Infrared (FTIR) technique to understand changes in the chemical structure in *Padina tetrastromatica* (Hauck). The marine brown alga under study was grown in two different environmental conditions; in natural seawater (*P. tetrastromatica* (c)) and in seawater suplemented with 50 ppm of cadmium (*P. tetrastromatica* (t)) for a three-week period in the laboratory. The second derivative, IR specrum in the mid-infrared region (4000–400 cm^−1^) was used for discriminating and identifying various functional groups present in *P. tetrastromatica* (c). On exposure to Cd, *P. tetrastromatica* (t) accumulated 412 ppm of Cd and showed perturbation in the band structure in the mid-IR absorption region. Variation in spectral features of the IR bands of *P. tetrastromatica* (untreated and treated) suggests that cadmium ions bind to hydroxyl, amino, carbonyl and phosphoryl functionalities. This was attributable to the presence of the following specific bands. A band at 3666 cm^−1^ in untreated *P. tetrastromatica* (c) while a band at 3560 cm^−1^ in Cd-treated *P. tetrastromatica* (t) due to non bonded and bonded O-H respectively. Similarly, non bonded N-H for *P. tetrastromatica* (c) showed two bands at 3500 cm^−1^ and 3450 cm^−1^ due to the N-H stretching vibrations and a band at 1577 cm^−1^ due to N-H bending vibrations, while an intense band at 3350 cm^−1^ due to bonded N-H stretching vibrations and at 1571 cm^−1^ due to bending vibrations was observed for Cd-treated *P. tetrastromatica* (t). Involvement of ester carbonyl group is characterized by the presence of a band at 1764 cm^−1^ in untreated *P. tetrastromatica (*c) while the Cd-treated *P. tetrastromatica* (t) showed the band at 1760 cm^−1^. The intensity of the band at 1710 cm^−1^ in the control samples decreased drastically after cadmium treatment indicating carbonyl of COOH to be involved in metal chelation. A band at 1224 cm^−1^ for untreated *P. tetrastromatica* (*c*) and at 1220 cm^−1^ for Cd-treated *P. tetrastromatica* (t) is indicative of the involvement of phosphoryl group in metal binding. Several other such changes were also evident and discussed in this paper. Based on our observation, FTIR technique proves to be an efficient tool for detecting structural changes and probable binding sites induced by the presence of a metal pollutant, cadmium, in the marine environment.

## Introduction

In the recent past, there is growing awareness about the metal pollutants in the marine environment (Hutton and Symon, 1986) and an equal concern about the same due to their accumulation in tissues of various organisms and finally their bio-magnification through the food chain (Murata et al. 1970; Drash, 1993). The main anthropogenic sources of heavy metals are various industrial point sources, including mining activities, foundries and smelters (Forstner and Wittman, 1981; Nriagu and Pacyna, 1988). Lead, Cadmium and Mercury are the three most polluting contaminants which, according to the U.S. Environmental Protection Agency (EPA), pose the greatest threat to the environment (ATSDR, 1999).

Metal remediation through common physico-chemical techniques is expensive and not ecofriendly hence, biotechnological approaches have received a great deal of attention as an alternative in the recent years. Application of microbial cells (bacteria, fungi, microalgae etc) for the same have been elaborately reviewed by Malik (2004). In addition to microbial cells, seaweeds are also known to have the ability to sequester heavy metals from the surrounding seawater (Ragan et al. 1979; Amado Filho et al. 1997; Figueira et al. 1999; Davis et al. 2000). The passive removal of toxic metals by brown algae has been beautifully reviewed by Davis et al. (2003). According to them, the ability of seaweeds to adsorb and accumulate metals mainly depend upon its chemical composition. Using live algae for the same purpose is also studied by Sobhan and Sternberg (1999) who are of the opinion that using live algae could be more advantageous since several algae have simple growth requirements and faster growth rate which can be easily grown to provide a regenerating supply of metal-removal material. Since it is well documented that brown algae, *Padina* spp. are able to withstand long term uptake of trace metals (Murugadas et al. 1995; Engdahl et al. 1998; Amado Filho et al. 1999; Sheng et al. 2004) and also because of its ubiquitous occurrence and abundance along the Indian coast, we chose *Padina tetrastromatica* (Hauck) (Dictyotales, Phaeophyta) to study the compositional changes and the probable binding sites of Cd using FTIR technique.

The algal samples were studied using Fourier Transform Infrared (FTIR) technique. During the last decade, Fourier Transform Infrared (FTIR) spectroscopy has proven and accepted to be a powerful tool for the study of biological samples (Dumas and Miller, 2003). The primary reason for this is that common biomolecules such as nucleic acids, lipids and carbohydrates, have characteristic functional groups having unique molecular vibrational modes (vibrational fingerprints) corresponding to specific infrared light frequencies (Griffiths and de Haseth, 1986). The composition and structure of molecular functional groups can be determined by analyzing the position, width, and intensity of infrared light absorption (Jackson and Mantsch, 1996; Yee et al. 2004). Morikawa and Senda were the first to utilize FTIR to study divalent cation binding to cell walls through pectic polysaccharides and carboxyl groups on algal cell wall in 1974 (Morikawa and Senda, 1974; Morikawa et al. 1974). Other important work included those of Schwarzmaier and Brehm, 1975; Homble et al. 1989; Homble et al. 1990; Drake et al. 1996; Pagnanelli et al. 2000 etc.

Since, the chemical composition of brown algae is well understood the study aims at visualizing the biochemical changes occurring in *P. tetrastromatica* (Hauck) when grown in natural seawater and in seawater supplemented with cadmium. This paper particularly aims at adopting metabolic fingerprinting through the use of Fourier Transform Infrared (FTIR) technique to demonstrate the difference in the chemical nature of a marine brown alga, *P. tetrastromatica* (Hauck). The results obtained are discussed in detail in the present study.

## Materials and Methods

### Algal material

*Padina tetrastromatica* (Hauck) was collected along with the substratum without disturbing the holdfast, from Anjuna (15°51’N; 73°52’E), Goa Coast, India. Collection was made by snorkeling and transported to the laboratory in polyethene containers containing seawater. On reaching the laboratory, the algal samples were freed from epiphytes and washed using seawater. They were next acclimatized in the laboratory under controlled conditions (25 °C ± 2 °C, 16 h/8 h light and dark cycle, pH 7.4) with constant aeration for a week. This one-week-old *P. tetrastromatica* (Hauck) was used in the experiment.

### Experimental setup

An initial experiment was conducted by growing the algae in tanks containing varying concentration of Cadmium (10, 25, 50, 75 and 100 ppm). Normal growth was observed in tanks containing up to 50 ppm of cadmium. At 75 ppm of cadmium a white envelope was seen growing over the alga indicating stressed growth while at 100 ppm, the alga showed a color change to dark-brown and finally decayed in the tank. Hence the present experiment was conducted as described below using 50 ppm of cadmium.

Four aquarium glass tanks containing 10 L of seawater each, from the collection site were used as control tanks and an identical set of tanks (4 nos.) containing seawater supplemented with 50 ppm of Cd (added as Cadmium chloride) were used as experimental tanks. Laboratory-acclimatized *P. tetrastromatica* (Hauck) was introduced into both the sets of tanks in quadruplets and aerated for 2 days. Seawater in both the control and experimental tanks were changed after every 2 days with fresh seawater. The concentration of Cd in seawater each time (in experimental tanks) was maintained at 50 ppm by renewing seawater containing the same concentration of Cd and the pH was adjusted to 6.7 as adsorption of cadmium was most favorable at pH between 5–7 (Upatham et al. 2002). The tanks were well aerated throughout the course of the experiment (3 weeks). At the end of 3 weeks, only whole, healthy and adult plants were harvested. Algal samples were initially rinsed in seawater followed by distilled water. Thereafter, the loosely adhered cadmium from the sample surface was desorbed by treating the samples with 10 mM EDTA solution followed by washing them with distilled water to remove excess EDTA (Wehrheim and Wettern, 1994). Both control and experimental samples were placed separately on blotting papers and then freeze dried in a lyophilizer for 8 hours using a lyophilizer (Heto DRYWINNER, Denmark) to ensure complete removal of moisture. The algal samples were later powdered and stored in a vacuum desiccator in airtight containers until further analysis.

### Analysis for metal concentration

Known weight (500 mg dry weight) of both control and cadmium treated dried algal powder was digested in Teflon bombs in nitric acid. The samples were digested for 2 hours in an oven at 100 °C. The resulting solution was made to 25 ml using distilled water and the concentration of Cd was measured using flame Atomic Absorption Spectrometry (AAS), Analyst 100, Perkin Elmer.

### Sample preparation for FTIR Spectroscopy

Sample preparation was carried out as described by Naumann et al. (1991b). Briefly, known weight of dry algal sample (1 mg) was taken in a smooth agate mortar and mixed thoroughly with 2.5 mg of dry potassium bromide (KBr) using a pestle. The powder was filled in the micro-cup of 2 mm internal diameter to obtain the diffuse reflectance infrared spectrum for replicate samples. All IR spectra were recorded at room temperature (26 °C ± 1° C) in the mid infrared range (4000–400 cm^−1^) using FTIR-8201PC, Fourier Transform Infrared Spectrometer (Shimadzu). Typically, 20 scans were signal-averaged for a single spectrum. Each spectrum is displayed in terms of absorbance as calculated from the reflectance-absorbance spectrum using the Hyper-IR software. To minimize the difficulties arising from unavoidable shifts, baseline correction was applied. Each spectrum was normalized as normalization produces a spectrum in which maximum value of absorbance becomes 2 and minimum value 0. To improve the resolution of complex bands, the digitized original spectrum was smoothed on noisy spectrum using Kubelka Munk algorithm and converted into its second derivative using the Savitzky-Golay algorithm (Savitzky and Golay, 1964) using 21-point smoothing.

In the course of this study, *P. tetrastromatica* (Hauck) from control tanks will be referred to as *P. tetrastromatica* (c) and Cd-treated *P. tetrastromatica* will be referred to as *P. tetrastromatica* (t).

## Results and Discussion

Laboratory acclimatized algal samples of *P. tetrastromatica* were analysed for metal cadmium content using AAS. The control samples showed undetected concentration of Cd. The experimental tanks containing Cd-treated *P. tetrastromatica,* accumulated 412 ppm of Cd which was almost 8 times its concentration in seawater. Algae are capable of accumulating heavy metals to concentrations, several order of magnitude higher than in surrounding medium (Amando Filho et al. 1997).

According to Davis et al. (2003), the property of sequestering metal contaminants from the marine environment by brown algae depends largely upon the chemical makeup of brown algae. Biosorption by algae is mainly attributed to the cell wall properties where both electrostatic attraction and complexation play a major role. Laminaran is the main storage product in brown algae (Percival and McDowell, 1967), while the cell wall comprises mainly of cellulose, alginic acid, and sulfated mucopolysaccharides called fucoidan (Kreger, 1962; Percival and McDowell, 1967; Smidsrod and Draget, 1996).

**Figure 1.**
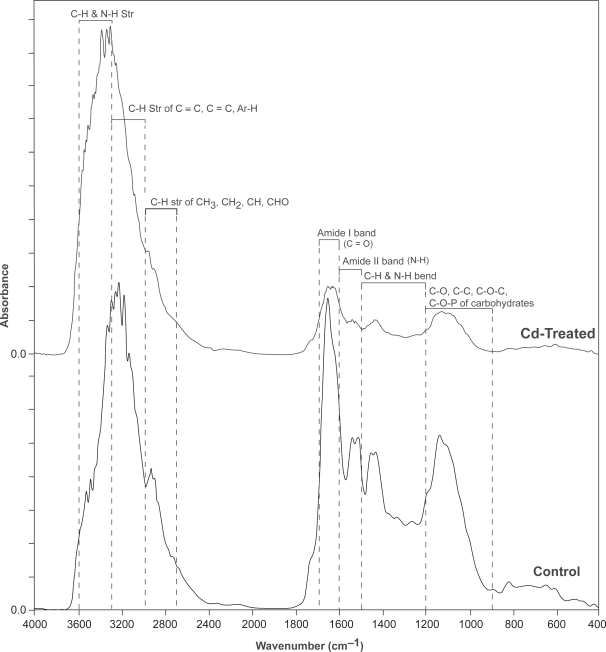
Comparison of the FTIR spectra of control *P. tetrastromatica* (c) and Cd-treated *P. tetrastromatica* (t) in the mid-infrared region (4000–400 cm^−1^). Characteristic functional groups contributing to the formation of absorption bands at specific wavenumbers are indicated in the figure.

The representative IR spectra from replicate studied in the mid-infrared region (4000–400 cm^−1^) for control *P. tetrastromatica* (c) and Cd-treated *P. tetrastromatica* (t) is as shown in Figure 1. Characteristic functional groups contributing to the formation of absorption bands at specific wave-numbers are indicated in the same figure (Fig. 1). In response to Cd stress, there was a general decrease in the protein and carbohydrate content indicated by a decrease in the intensity of absorption bands especially in the 1800 to 800 cm^−1^ region. This region is specific for proteins and carbohydrates (Dumas and Miller, 2003; Wolkers et al. 2004; Yee et al. 2004). Several other researchers have also used similar FTIR technique for studying changes in the chemical composition in higher plants and algae (Adhiya et al. 2002; Dokken et al. 2002; Sheng et al. 2004).

**Figure 2.**
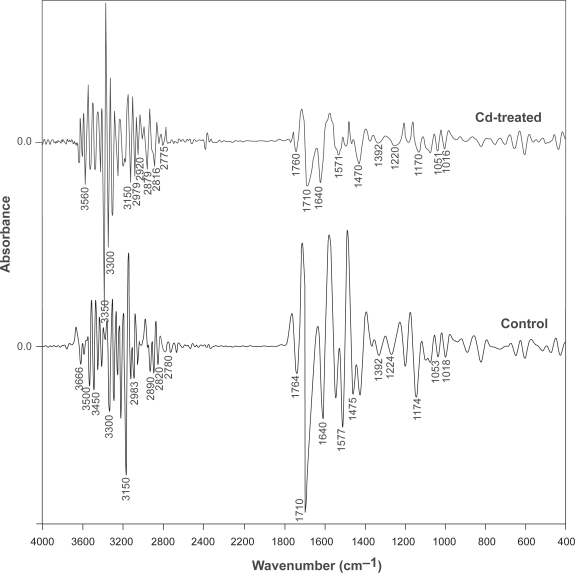
Comparison of the second derivative spectra of control *P. tetrastromatica* (c) and Cd-treated *P. tetrastromatica* (t) in the mid-infrared region (4000–400 cm^−1^).

However, as can be seen in Figure 1, extensive overlapping of bands in the raw spectrum causes difficulty in band differentiation and their assignment. Hence, using raw spectrum in the interpretation of data may not be totally conclusive, since the raw data obtained are often noisy (Sasic et al. 2005). Thus, in the present study, we resolved this issue by treating the raw spectrum with Kubelka Munk algorithm and later converting into the second derivative data for further interpretation. The second derivative IR spectra for both the control and Cd treated algal culture for discriminating and identifying various functional groups is shown in Figure 2.

An advantage of this method is that the data is de-noised to a great extent and overlapping is largely minimized. Comparison of the second derivative IR spectra for control and Cd-treated *P. tetrastromatica* (t) causes visualization in the band structure perturbation more distinctly (Fig. 2).

Table 1 shows the assignment of different bands to distinct functional groups that belong to different class of macromolecules in the control samples as well as perturbation in the band structure after Cd-treatement.

Careful examination of the second derivative IR spectra for both *P. tetrastromatica* (c) and *P. tetrastromatica* (t) revealed several differences (Fig. 2). The region 3700–3300 cm^−1^ is characteristic for O-H and N-H stretching vibrations (Guo and Zhang, 2004). The IR spectrum of *P. tetrastromatica* (c) showed a band at 3666 cm^−1^ while a band at lower wavenumber (3560 cm^−1^) was observed for *P. tetrastromatica* (t). This is mainly due to free (non-bonded) O-H stretching vibrations in the former spectrum as compared to the bonded O-H in the latter (Blokker et al. 1998; Sigee et al. 2002). On treatment with Cd, the hydroxyl group gets involved in metal-oxygen binding as evident by the band shift to lower wavenumber, indicating the role of hydroxyl group in metal bonding. A similar situation is also evident for explaining the role of NH group bonding with Cd. Control *P. tetrastromatica* (c) showed the presence of two medium sized bands at wavenumbers 3500 cm^−1^ and 3450 cm^−1^ due to N-H stretching vibrations (non-bonded) of primary amine (Sigee et al. 2002) and a band at 1577 cm^−1^ due to N-H bending vibrations (Sigee et al. 2002; Fischer et al. 2006). On treatment with Cd, there was only one intense band due to N-H stretching at 3350 cm^−1^ while the N-H bending vibrations caused the band to appear at 1571 cm^−1^ (Fig. 2). These spectral features explains two points, firstly, the presence of a primary amine in *P. tetrastromatica* (c) which gets converted into a secondary amine after treatment with Cd. Secondly, chelation of Cd with NH functionality resulted in weakening of the N-H bond strength converting it into a bonded NH group and causing a downshift of the stretching as well as the bending absorption bands. According to Golcuk et al. (2003), the result of binding with the metal Cd causes alteration of the hybridization type around nitrogen causing weakening of NH bond.

Thus, non-bonded O-H and N-H are transformed into bonded O-H and N-H when *P. tetrastromatica* was grown in the presence of Cd. Previous studies suggest hydroxyl groups to have high affinity for divalent cations (Fourest et al. 1994; Puranik et al. 1999). These hydroxyl groups especially present in all polysaccharides can become negatively charged thereby contributing to metal adsorption to a significant level (Forsberg, 1988).

The region 3300 to 3000 cm^−1^ is characteristic for C-H stretching vibrations of C ≡ C, C = C and Ar-H, while the region from 3000 to 2700 is dominated by the C-H stretching vibrations of −CH_3_, >CH_2_, CH and CHO functional groups respectively (Stuart, 1997; Dumas and Miller, 2003). Two bands were identified at wavenumbers 3300 and 3150 cm^−1^ in *P. tetrastromatica* (c) which were also present in *P. tetrastromatica* (t). In addition, bands at wavenumbers 2983, 2890, 2820 and 2780 cm^−1^ were evident in *P. tetrastromatica* (c) where as in *P. tetrastromatica* (t) this region showed bands at wavenumbers 2979, 2920, 2879, 2816 and 2775 cm^−1^ respectively. An additional band at 2920 cm^−1^ was observed in *P. tetrastromatica* (t) when compared to *P. tetrastromatica* (c) which was accountable for additional asymmetric C-H stretching vibrations of methylene (Stuart, 1997) in the former as compared to the latter (Table 1). All the other bands in this region showed a shift to lower wavenumber in Cd-treated *P. tetrastromatica* (t).

The region between 1800 and 1500 cm^−1^ show characteristic bands for proteins, wherein 1700 to 1600 cm^−1^ is specific for amide-I bands (Dumas and Miller, 2003), which is mainly due to C = O stretching vibrations of peptide bond (Backmann et al. 1996). The bands in the amide I region provide insight into the protein secondary structure (Byler and Susi, 1986). On the other hand the region from 1600 to 1500 cm^−1^ is specific for amide-II bands, which is due to N-H bending vibrations (Fischer et al. 2006). IR spectrum of *P. tetrastromatica* (c) showed a band at 1764 cm^−1^ which is due to C = O stretching of ester carbonyl (Fischer et al. 2006) which shifted to 1760 cm^−1^ in Cd-treated *P. tetrastromatica* (t). The intensity of the absorption band at 1710 cm^−1^ in control *P. tetrastromatica* (c) decreased considerably in *P. tetrastromatica* (t) and is nearly the same as the chelated carboxyl at 1640 cm^−1^ as is evident in the spectrum in Figure 2. This is indicative that the carboxyl of COOH is utilized for chelation. The band at 1577 cm^−1^ in *P. tetrastromatica* (c) showed a down shift to 1571 cm^−1^ in *P. tetrastromatica* (t), which is due to N-H bending vibrations (Naumann et al. 1996; Fischer et al. 2006). This non-invasive method has been applied to detect changes of the overall protein secondary structure during dehydration in maize (Zea mays) embryo (Walkers et al. 1998) and pollen (Wolkers and Hoekstra, 1995)

The bands in the 1500–1200 cm^−1^ region arise mainly from the C-H bending vibrations of CH_3_, CH_2_ and CH functional groups (Wolkers et al. 2004; Yee et al. 2004). Information on phosphodiester functional groups can be obtained in the region between 1250 and 1200 cm^−1^ which corresponds to > P = O asymmetric stretching frequencies (Dumas and Miller, 2003; Yee et al. 2004). In the present study, *P. tetrastromatica* (c) showed an absorption band at 1224 cm^−1^ while *P. tetrastromatica* (t) showed the band at 1220 cm^−1^ which is due to P = O asymmetric stretching vibrations of PO_2_− phosphodiesters. Phosphate moieties adopt chelating properties and among the phosphate containing metabolites, sugar phosphate esters are known to play a crucial role in metal chelation (Fiona et al. 2003).

The region from 1200 to 900 cm^−1^ are mainly dominated by a sequence of bands due to C-O, C-C, C-O-C and C-O-P stretching vibrations of polysaccharides (Wolkers et al. 2004; Yee et al. 2004) as well as CH_3_, CH_2_ rocking modes (Wolpert and Hellwig, 2006). These groups mainly occur in carbohydrates and cellular polysaccharides. *P. tetrastromatica* (c) showed polysaccharide bands at 1174, 1053, and 1018 cm^−1^ (Fig. 2 control). On the other hand, *P. tetrastromatica* (t) exposed to Cd showed bands at 1170, 1051, and 1016 cm^−1^ (Fig. 2 Cd-treated). Bands in the region 1200–900 cm^−1^ showed changes in their position towards lower wavenumber upon sugar-cadmium metalation. Because of the complexity of the absorption of various cellular polysaccharides, specific assignments are rather difficult. Although, it was earlier speculated that carbohydrates play a significant role in metal adsorption, in addition to this, variation in spectral features attributes to hydroxyl, carbonyl, amino and phosphoryl groups to play a significant role in metal adsorption. Further study is on to see if other members of brown algae also behave in a similar manner.

In conclusion, Fourier Transform Infrared (FTIR) technique has been successfully used as a tool to study the stress-induced changes occurring in *P. tetrastromatica* (Hauck) when grown in two different environmental conditions. Although, there was no visible morphological change in the *P. tetrastromatica* from both the control and experimental tanks, perturbations in the IR band structure indicated chemical alterations in *P. tetrastromatica* (t) when exposed to 50 ppm of Cd. Comparison of IR data of both control and Cd-treated *P. tetrastromatica* provided direct evidence to show that hydroxyl, carbonyl, amino and phosphoryl functionalities played a significant role in metal chelation. The non-bonded hydroxyl groups on metal-oxygen interaction showed characteristic bonded O-H stretching vibrations in Cd-treated samples. So also was the case with amine group. The N-H bond strength weakens upon coordination with Cd and causes a downshift of the non bonded N-H band in *P. tetrastromatica* (c) converting it into a bonded N-H group in *P. tetrastromatica* (t). Thus, non-bonded O-H and N-H are transformed into bonded O-H and N-H when *P. tetrastromatica* was grown in the presence of Cd. Other important changes worth mentioning is that of phosphate moieties adopting chelating properties. Phosphate-containing metabolites, probably the sugar phosphate esters (from the cell wall) seem to play a crucial role by interacting with Cd as indicated by band shift in the IR spectrum. Another interesting feature is the shift in ester carbonyl bands and decrease in the intensity of the acid carbonyl bands, also indicating their role in metal chelation.

Hence, this technique is a reliable and efficient tool for studying stress-induced changes in a marine alga *P. tetrastromatica* under cadmium pollution. This technique is sensitive and is particularly important when a large number of samples need to be analyzed in a short period of time as it involves minimum sample preparation steps and requires very small quantity (<2 mg) of sample to yield IR spectrum. Hence, it is possible to identify the broad chemical composition of biological samples using FTIR which uses a non-invasive extraction-less method.

## Figures and Tables

**Table 1.** Band (peak) assignments for second derivative infrared spectra.

***P. tetrastomatica* (Control)**		***P. tetrastomatica* (Cd-Treated)**		**Functional group assignment (Reference)**
**Peak no.**	**Wavenumber = (cm^−1^)**	**Peak no.**	**Wavenumber = (cm^−1^)**	
1	3666	1	3560	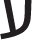 _s_ O-H (Guo and Zhang, 2004)
2,3	3500, 3450	2	3350	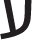 _s_ N-H (Sigee et al. 2002)
4	3300	3	3300	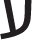 _s_ C-H of C ≡ C, C = C and Ar-H (Sigee et al. 2002)
5	3150	4	3150	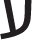 _s_ C-H of C ≡ C, C = C and Ar-H (Sigee et al. 2002)
6	2983	5	2979	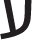 _s_ C-H of CH_3_, CH_2_, CH (Dumas and Miller, 2003)
		6	2920	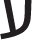 _s_ C-H of CH_3_, CH_2_, CH (Dumas and Miller, 2003)
7	2890	7	2879	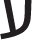 _s_ C-H of CH_3_, CH_2_, CH (Dumas and Miller, 2003)
8	2820	8	2816	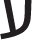 _s_ C-H of CH_3_, CH_2_, CH (Dumas and Miller, 2003)
9	2780	9	2775	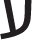 C-H of CHO (Stuart, 1997)
10	1764	10	1760	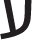 _s_ C = O of ester group (Fischer et al. 2006)
11	1710	11	1710	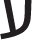 C = O of COOH (Naumann et al. 1996; Garcia et al. 2005)
12	1577	12	1571	δ N-H of primary amines (Fischer et al. 2006)
13	1475	13	1470	δ_s_ C-H of CH_3_, CH_2_ groups (Wolkers et al. 2004; Yee et al. 2004)
14	1392	14	1392	δ_s_ C-H of CH_3,_ CH_2_ groups and _s_ C-O of COO^−^ groups (Giordano et al. 2001)
15	1224	15	1220	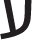 _as_ P = O of phosphodiester group of nucleic acids and phospholipids (Dumas and Miller, 2003; Yee et al. 2004)
16	1174	16	1170	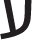 _s_ C-C, C-O, C-O-C and C-O-P of saccharides (Wolkers et al. 2004; Yee et al. 2004)
17	1053	17	1051	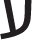 _s_ C-C, C-O, C-O-C and C-O-P of saccharides (Wolkers et al. 2004; Yee et al. 2004)
18	1018	18	1016	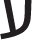 _s_ C-C, C-O, C-O-C and C-O-P of saccharides (Wolkers et al. 2004; Yee et al. 2004)

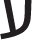
, stretching;

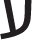
_s_, symmetric stretching;

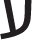
_as_, asymmetric stretching;

δ, bending;

δ_s_, symmetric bending.
